# Genetic associations of *visfatin* polymorphisms with clinicopathologic characteristics of prostate cancer in Taiwanese males

**DOI:** 10.7150/ijms.101020

**Published:** 2024-09-23

**Authors:** Sung-Lin Hu, Shan-Chi Liu, Chia-Yen Lin, Yi-Chin Fong, Shian-Shiang Wang, Li-Chai Chen, Shun-Fa Yang, Chih-Hsin Tang

**Affiliations:** 1 School of Medicine, China Medical University, Taichung, Taiwan.; 2 Department of Family Medicine, China Medical University Hsinchu Hospital, Hsinchu, Taiwan.; 3 Institute of Biomedical Sciences, Mackay Medical College, New Taipei City, Taiwan.; 4 Division of Urology, Department of Surgery, Taichung Veterans General Hospital, Taichung, Taiwan.; 5 School of Medicine, Chung Shan Medical University, Taichung, Taiwan.; 6 School of Medicine, National Yang Ming Chiao Tung University, Taipei, Taiwan.; 7 Department of Sports Medicine, College of Health Care, China Medical University, Taichung, Taiwan.; 8 Department of Orthopedic Surgery, China Medical University Hospital, Taichung, Taiwan.; 9 Department of Orthopedic Surgery, China Medical University Beigang Hospital, Yunlin, Taiwan.; 10 Department of Applied Chemistry, National Chi Nan University, Nantou, Taiwan.; 11 Department of Pharmacy and Master Program, Collage of Pharmacy and Health Care, Tajen University, Pingtung County, Taiwan.; 12 Institute of Medicine, Chung Shan Medical University, Taichung, Taiwan.; 13 Department of Medical Research, Chung Shan Medical University Hospital, Taichung, Taiwan.; 14 Department of Pharmacology, School of Medicine, China Medical University, Taichung, Taiwan.; 15 Department of Medical Laboratory Science and Biotechnology, Asia University, Taichung, Taiwan.; 16 Chinese Medicine Research Center, China Medical University, Taichung, Taiwan.; 17 Department of Medical Research, China Medical University Hsinchu Hospital, Hsinchu, Taiwan.

**Keywords:** Prostate cancer, Single nucleotide polymorphism, Visfatin, Taiwanese males

## Abstract

The most general cancer in men is prostate cancer (PCa), with its risk increasing due to age and obesity. Visfatin, a member of adipokines, is related to cancer progression and metastasis, but its relationship in PCa remains undetermined. In addition, no knowledge is available regarding relations between *visfatin* polymorphisms and clinicopathological characteristics in PCa. We sought to investigate the functions of four *visfatin* gene polymorphisms and clinicopathological characteristics on the hazard of developing PCa in 695 Taiwanese males with PCa. Carriers of the GA+AA heterozygote of SNP rs61330082 were at a markedly higher risk of biochemical recurrence than those with the GG genotype. *Visfatin* rs61330082 and rs11977021 were related with a high risk of perineural invasion, lymphovascular invasion, and biochemical recurrence in prostate-specific antigen (PSA) > 10 PCa patients. The Cancer Genome Atlas database noted that *visfatin* mRNA level did not prominently differ with pathological T/N stage and overall survival. This finding is the first to document a connection between *visfatin* polymorphisms and clinicopathological characteristics of PCa in Taiwanese males.

## Introduction

The most general cancer in men is prostate cancer (PCa), with its risk increasing due to age and obesity 1-3. Projections estimate 300,000 new patients of PCa in the US by 2050. While many initially present with localized disease, over half of PCa patients eventually progress bone metastases, particularly in advanced stages 2, 4, 5. Despite a 98% five-year associate survival rate for combined diagnosed cases of PCa, numerous patients with metastatic cancer face treatment-resistant disease. PCa metastases can significantly reduce patients' quality of life and are a prominent cause of death 6, 7. A solid comprehension of the mechanisms driving malignant development and distant metastasis in PCa would facilitate the evolution of early diagnosis and prevention managements.

An expanding body of research suggests a connection between cancer and obesity 8. This link is particularly evident in cancers such as esophageal, pancreatic, and colorectal cancers 8, 9. Furthermore, obesity can alter the cancer's microenvironment, accelerating its progression 10. Visceral fat contains substantial amounts of visfatin, an adipokine that promotes inflammation 11-13. Visfatin is known to regulate several cellular functions in mammalian cells, including cellular proliferation, migration, differentiation, and apoptosis 11. It is not surprising that individuals with various malignancies exhibit significantly higher visfatin levels compared to people without cancer 14, 15. Visfatin expression is recognized as vital in various tumor-associated activities, including survival, angiogenesis, metastasis, and treatment resistance 16. Reports have demonstrated that visfatin associates PCa growth and survival 17, 18.

The *visfatin* gene's promoter, 5'- and 3'-untranslated regions (UTRs), have more than 150 single nucleotide polymorphisms (SNPs), and it is thought that this significant variability controls visfatin function and output 19. *Visfatin* gene polymorphisms have been linked to hepatocellular and esophageal squamous cell carcinoma 20, 21. Our lab's earlier research detailed the relationship between several *visfatin* polymorphisms and the likelihood of developing oral and lung cancer in Taiwanese individuals 22, 23. Our goal was to ascertain the relationships between four *visfatin* gene SNPs and clinicopathological characteristics associated with the risk of PCa in Taiwanese males. We believe that this is the first report in a Taiwanese population to demonstrate a substantial correlation between *visfatin* polymorphisms and PCa.

## Materials and Methods

### Participants

From 2012 to 2018, Taichung Veteran General Hospital treated 695 patients with adenocarcinoma of the prostate who underwent robot-assisted radical prostatectomy with bilateral standard pelvic lymph node dissection. Prior to the start of the trial, each participant provided informed written consent, and the Taichung Veteran General Hospital's Institutional Review Board (IRB) accepted the protocol (IRB No. CE19062A). The initial prostate-specific antigen (PSA) at diagnosis, clinical and pathological tumor-node-metastasis (TNM) staging, Gleason score of the initial biopsy, D'Amico classification 24, Gleason grade group, and other pathological features from the permanent pathological document 25 were obtained from the medical records of the patients. The eighth edition of the American Joint Committee on Cancer Staging Manual's TNM staging system was used to stage PCa patients. Our patient grouping consisted of 331 individuals with PSA level of less than 10 ng/ml (PSA ≤ 10 group) and 364 individuals with PSA level greater than 10 ng/ml (PSA > 10 group).

### Genomic DNA extraction and PCR genotyping

Genomic DNA was isolated from peripheral blood using a QIAamp DNA Blood Kit (Qiagen, CA, USA) according the manufacturer's procedures 26. Allelic discrimination for *visfatin* SNPs was analyzed following the manufacturer's procedures, as described in our previous studies 22, 23. The promoter region contains rs11977021, rs61330082, and rs2110385, three of the four *visfatin* SNPs that were analyzed. Between exons six and seven, in the intron region, is where *visfatin* rs4730153 is found. The TaqMan SNP Genotyping Assay and the ABI StepOnePlus Real-Time PCR system (Applied Biosystems, Foster City, CA, USA) were used to perform allele discrimination of four *visfatin* SNPs: C/T alleles of rs11977021, G/A alleles of rs61330082, G/T alleles of rs2110385, and G/A alleles of rs4730153. The procedure was followed as previously reported 22, 23. The *visfatin* SNP probes rs11977021 (C_11405260_10), rs61330082 (C_88870749_10), rs2110385 (C_16125685_10) and rs4730153 (C_2673294_10) was purchased from Applied Biosystems (Foster City, CA, USA).

### Analysis of clinical dataset

Levels of *visfatin* in PCa samples collected from The Cancer Genome Atlas (TCGA) via the UALCAN website (https://ualcan.path.uab.edu/) were analyzed 3, identifying 482 patients whose *visfatin* gene expression was measured in each tumor sample.

### Statistical analysis

Data were analyzed using the statistical software program Statistical Analytic System version 9.1 (SAS Institute Inc., Cary, NC, USA). Chi-square and Student's t-test were employed to compare demographic characteristics between the PSA ≤ 10 and > 10 groups. Odds ratios (ORs) and adjusted ORs (AORs) with 95% confidence intervals (CIs) were estimated using multiple logistic regression models to determine the association between genotypic frequencies and the two PSA groups, as well as the risk of different clinicopathological characteristics. Between-group differences were considered significant when *p*-values were <0.05.

## Results

The blood level of PSA is often elevated in individuals with PCa, serving as a marker to monitor disease progression 27. This study recruited 695 Taiwanese males (Table 1), compared with the PSA ≤ 10 group, the PSA > 10 group had markedly more patients aged > 65 years, pathological Gleason grade group 4 + 5, clinical and pathologic T3 + 4, and a higher incidence of clinical M1 disease, pathologic N1, seminal vesicle invasion, perineural invasion, and lymphovascular invasion. Pathological examination revealed lymph node metastasis rates of 4.5% and 12.4% in the PSA ≤ 10 and PSA > 10 groups. The percentages of high-risk PCa were 27.8% (92) in the PSA ≤ 10 group and 70.1% (255) in the PSA > 10 group, according to the D'Amico classification (Table 1).

Next, we examined the role of *visfatin* gene polymorphisms on clinicopathologic characteristics of PCa patients. Compared with having the GG genotype at rs61330082, having the GA+AA heterozygote markedly evaluated the risk of biochemical recurrence (OR 1.462; 95% CI, 1.013-2.110; *p*<0.05) (Table 3). We then examined the relation of clinicopathological characteristics in the PSA > 10 group, as shown in Tables 4 and 5. Compared with individuals carrying the GG genotype, those carrying the GA+AA genotype showed a higher incidence of pathologic N1 stage (OR 2.589; 95% CI, 1.118-5.994; *p*<0.05), perineural invasion (OR 1.948; 95% CI, 1.139-3.331; *p*<0.05), lymphovascular invasion (OR 1.960; 95% CI, 1.089-3.527; *p*<0.05), and biochemical recurrence (OR 1.755; 95% CI, 1.099-2.805; *p*<0.05) (Table 4).

Furthermore, among PSA > 10 group PCa patients with the SNP rs11977021, having the CT+TT heterozygote increased the risk of perineural invasion (OR 1.777; 95% CI, 1.038-3.041; *p*<0.05), lymphovascular invasion (OR 1.824; 95% CI, 1.024-3.249; *p*<0.05), and biochemical recurrence (OR 1.601; 95% CI, 1.007-2.545; *p*<0.05) compared with having the CC wild-type (Table 5).

**Figure 1:**
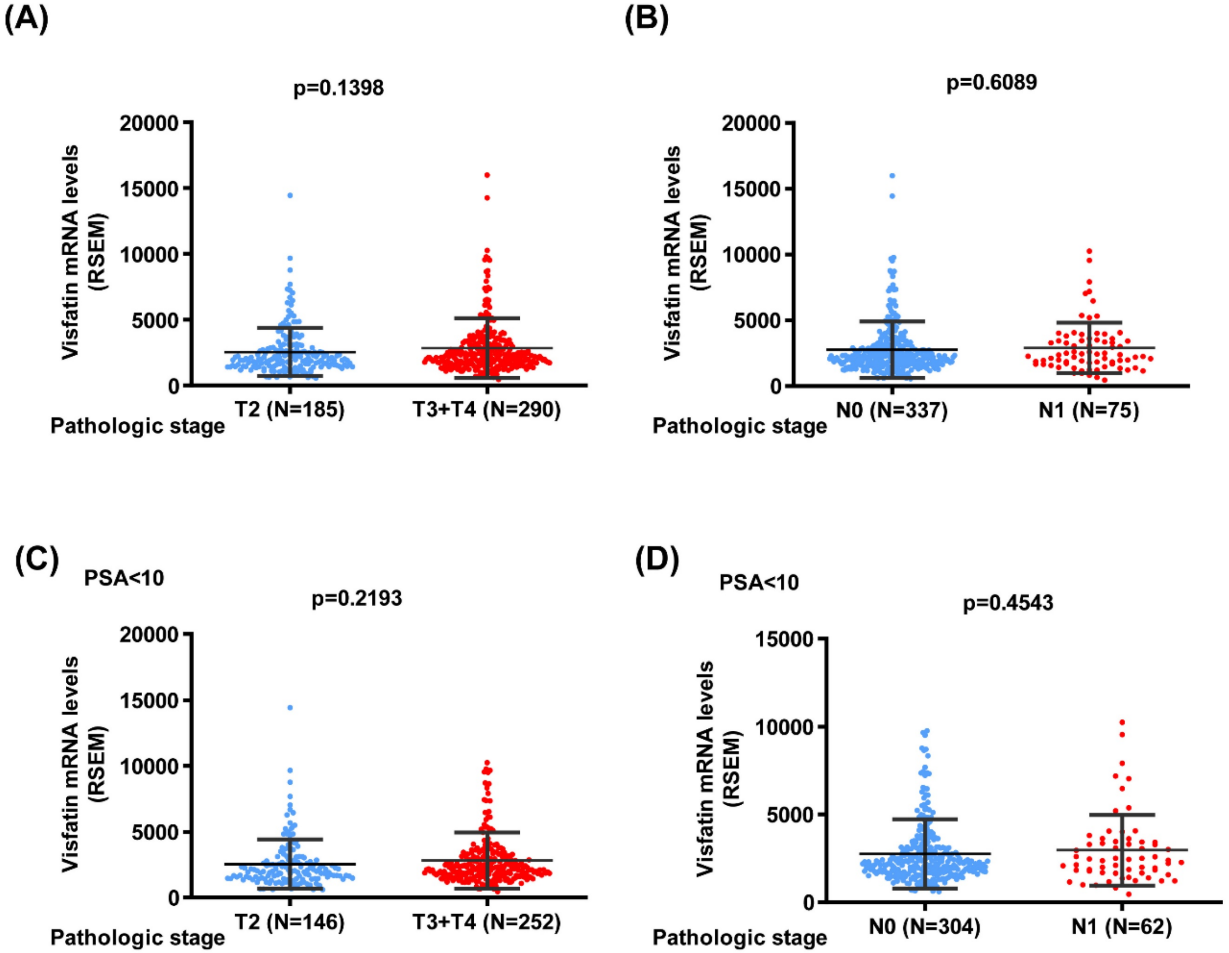
** The *visfatin* mRNA level of PCa patients from TCGA database.** Levels of *visfatin* mRNA expression in whole population (A&B) and PSA < 10 group (C&D) PCa patients retrieved from TCGA dataset records.

**Figure 2:**
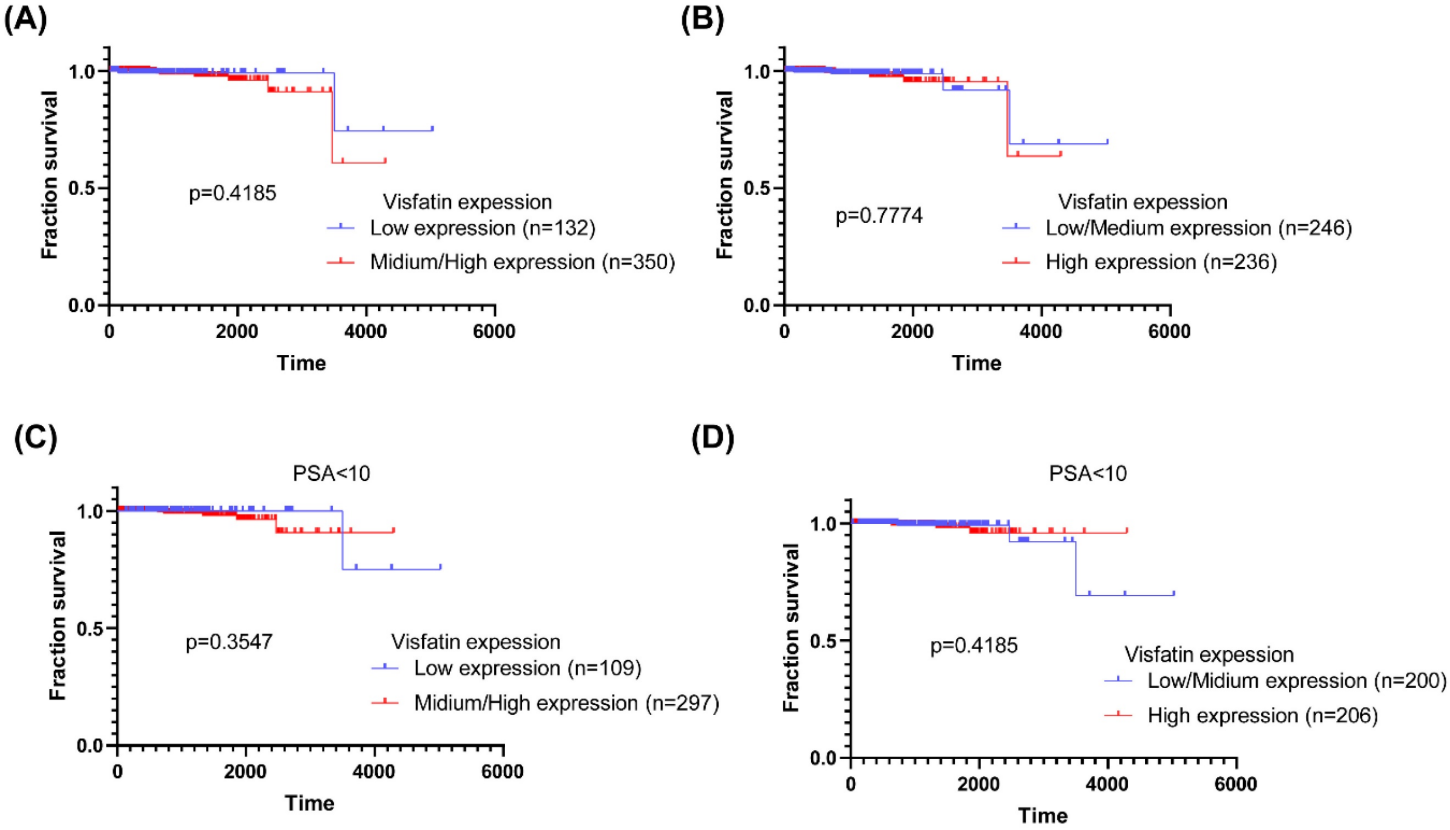
** The *visfatin* mRNA level and overall survival of PCa from the TCGA database.** Kaplan-Meier analysis examined levels of *visfatin* expression and overall survival rates of whole population (A&B) and PSA < 10 group (C&D) PCa patients.

We next used the TCGA database to analyze the *visfatin* mRNA level, PSA level at diagnosis, pathological T/N stage, and overall survival. No noteworthy difference was found between the T2 and T3 + 4 groups or pathological N stages of PCa in terms of *visfatin* level (Fig. 1A&B). Similar results were also observed in PCa patients with PSA levels ≤10 ng/ml (Fig. 1C&D). Additionally, no difference was observed in the overall survival between high and low *visfatin* level in the whole population group (Fig. 2A&B) and PSA levels ≤10 ng/ml group (Fig. 2C&D).

## Discussion

Adipose tissue plays a critical effect in PCa progression 28. PCa is believed to engage in direct or indirect interactions with adipocytes, influencing tumor cell growth, invasion, and metastasis. Notably, bone constitutes the most prevalent metastatic site in PCa 29, 30. Multiple reports have suggested bone marrow adipocytes as significant contributors to the development and aggravation of these bone metastases 31. Visfatin is strongly related to oncogenesis, and in many types of cancer, upregulated visfatin production are related with a worse prognosis 32-34. However, the TCGA database results for the entire population group showed no difference in overall survival between the groups with high and low *visfatin* mRNA expression. This lack of distinction might be attributed to the brief follow-up period and relatively small sample size. Further research is needed, necessitating an increase in both sample size and follow-up time.

In addition to the aforementioned adipocytokines, visfatin, an adipokine found in visceral fat, has been documented to regulate cancer development and metastasis in studies involving various cancers such as liver, endometrial, esophageal and osteosarcoma 35-38. However, its impact on the clinicopathologic characteristics of PCa patients has not yet been established. The TCGA database results for the current human PCa investigation showed that there was no statistically significant difference in *visfatin* mRNA levels between pathological T/N stage in PCa patients. However, compared with having the GG genotype at rs61330082, having the GA+AA heterozygote significantly increased the risk of biochemical recurrence, while no such association was observed with other clinicopathological characteristics. This evidence implicates crucial roles for *visfatin* polymorphisms in PCa biochemical recurrence.

Over the past ten years, PSA testing has gained popularity worldwide, and a single PSA screening intervention alone can increase the examination of low-risk PCa 39. Numerous genomic-based assays have been developed to predict metastasis, cancer-related mortality, and recurrence following surgery 40, 41. To ascertain predictive variables for active surveillance, several alleles were evaluated 42. This is the first time to assess the relation between *visfatin* SNPs and the clinicopathological features of PCa with iPSA levels > 10 ng/ml. The data indicated that individuals with the GA+AA genotype at *visfatin* rs61330082 had a high risk of pathologic N1 stage, perineural invasion, lymphovascular invasion, and biochemical recurrence in the PSA > 10 group. The *visfatin* SNP rs11977021, having the CT+TT heterozygote, increased the risk of perineural invasion, lymphovascular invasion, and biochemical recurrence in PSA > 10 group PCa patients.

The term 'tumor metastasis' refers to the process through which the primary tumor disseminates to diverse tissues or organs via the blood or lymphatic system. Given that restraining lymphangiogenesis can effectively impede both tumor progression and metastasis 43-45, comprehending the intricate nature of metastasis is of paramount importance. The link between lymphangiogenesis and tumor growth as well as metastasis has been substantiated through various scholarly publications 46, 47. Our results indicated that *visfatin* SNPs, rs61330082 and rs11977021, are associated with lymphovascular invasion in PSA > 10 group PCa patients. Therefore, *visfatin* SNPs in PSA > 10 group PCa patients can be used as prognostic factors for lymphangiogenesis.

In conclusion, our examination demonstrates associations between *visfatin* gene variants and biochemical recurrence for PCa, particularly among Taiwanese males carrying the *visfatin* rs61330082 polymorphism. Additionally, *visfatin* SNPs, rs61330082 and rs11977021, are associated with perineural invasion, lymphovascular invasion, and biochemical recurrence in PSA > 10 group PCa patients.

## Figures and Tables

**Table 1:** The distributions of demographical characteristics in 695 patients with prostate cancer.

Variable	PSA at diagnosis (ng/mL)		
	≤ 10 (n=331)	> 10 (n=364)	p value
**Age at diagnosis (years)**			
≤ 65	159 (48.0 %)	136 (36.6 %)	p=0.004*
> 65	172 (52.0 %)	228 (63.4 %)	
**Pathologic Gleason grade group**			
1+2+3	301 (90.9 %)	276 (75.8 %)	p<0.001*
4+5	30 (9.1 %)	88 (24.2 %)	
**Clinical T stage**			
1+2	311 (94.0 %)	286 (78.6 %)	p<0.001*
3+4	20 (6.0 %)	78 (21.4 %)	
**Clinical N stage**			
N0	328 (99.1 %)	353 (97.0 %)	p=0.047*
N1	3 (0.9 %)	11 (3.0 %)	
**Clinical M stage**			
M0	331 (100.0 %)	353 (97.0 %)	p=0.001*
M1	0 (0.0 %)	11 (3.0 %)	
**Pathologic T stage**			
2	227 (68.6 %)	139 (38.2 %)	p<0.001*
3+4	104 (31.4 %)	225 (61.8 %)	
**Pathologic N stage**			
N0	316 (95.5 %)	319 (87.6 %)	p<0.001*
N1	15 (4.5 %)	45 (12.4 %)	
**Seminal vesicle invasion**			
No	300 (90.6 %)	247 (67.9 %)	p<0.001*
Yes	31 (9.4 %)	117 (32.1 %)	
**Perineural invasion**			
No	113 (34.1%)	71 (19.5 %)	p<0.001*
Yes	218 (65.9 %)	293 (80.5 %)	
**Lymphovascular invasion**			
No	305 (92.1 %)	280 (76.9 %)	p<0.001*
Yes	26 (7.9 %)	84 (23.1 %)	
**Biochemical recurrence**			
No	264 (79.8 %)	211 (58.0 %)	p<0.001*
Yes	67 (20.2 %)	153 (42.0 %)	
**D'Amico classification**			
Low risk/ Intermediate risk	239 (72.2 %)	109 (29.9 %)	p<0.001*
High risk	92 (27.8 %)	255 (70.1 %)	

**Table 2:** Distribution frequency of *visfatin* genotypes in 695 patients with prostate cancer.

Variable	PSA at diagnosis (ng/mL)			
	≤ 10	> 10	OR (95% CI)	AOR (95% CI)
**rs11977021**				
CC	90 (27.2%)	111 (30.5%)	1.000 (reference)	1.000 (reference)
CT	174 (52.6%)	179 (49.2%)	0.834 (0.589-1.181)	0.777 (0.521-1.159)
TT	67 (20.2%)	74 (20.3%)	0.896 (0.581-1.379)	0.701 (0.425-1.155)
CT+TT	241 (72.8%)	253 (69.5%)	0.851 (0.612-1.183)	0.755 (0.517-1.102)
**rs61330082**				
GG	90 (27.2%)	110 (30.2%)	1.000 (reference)	1.000 (reference)
GA	173 (52.3%)	178 (48.9%)	0.842 (0.594-1.193)	0.767 (0.514-1.144)
AA	68 (20.5%)	76 (20.9%)	0.914 (0.595-1.405)	0.709 (0.432-1.165)
GA+AA	241 (72.8%)	254 (69.8%)	0.862 (0.620-1.199)	0.750 (0.513-1.095)
**rs2110385**				
GG	260 (78.5%)	297 (81.6%)	1.000 (reference)	1.000 (reference)
GT	64 (19.3%)	61 (16.8%)	0.834 (0.566-1.230)	0.847 (0.543-1.323)
TT	7 (2.1%)	6 (1.6%)	0.750 (0.249-2.261)	0.839 (0.248-2.844)
GT+TT	71 (21.5%)	67(18.4%)	0.826 (0.569-1.200)	0.847 (0.552-1.298)
**rs4730153**				
GG	262 (79.2%)	300 (82.4%)	1.000 (reference)	1.000 (reference)
GA	64 (19.3%)	59 (16.2%)	0.805 (0.545-1.190)	0.827 (0.529-1.294)
AA	5 (1.5%)	5 (1.4%)	0.873 (0.250-3.050)	0.854 (0.218-3.347)
GA+AA	69 (20.8%)	64 (17.6%)	0.810 (0.555-1.183)	0.829 (0.538-1.278)

The odds ratios (ORs) and with their 95% confidence intervals (CIs) were estimated by logistic regression models. The adjusted odds ratios (AORs) with their 95% confidence intervals (CIs) were estimated by multiple logistic regression models after controlling for Age at diagnosis, pathologic Gleason grade group, clinical T stage, clinical N stage, clinical M stage, pathologic T stage, pathologic N stage, seminal vesicle invasion, perineural invasion, lymphovascular invasion, biochemical recurrence and D'Amico classification.

**Table 3:** Odds ratio (OR) and 95% confidence interval (CI) of clinical status and *visfatin* rs61330082 genotypic frequencies in 695 patients with prostate cancer.

Variable	Genotypic frequencies			
**rs61330082**	**GG (N=200)**	**GA+AA (N=495)**	**OR (95% CI)**	**p value**
**Pathologic Gleason grade group**				
1+2+3	169 (84.5%)	408 (82.4%)	1.00	p=0.509
4+5	31 (15.5%)	87 (17.6%)	1.162 (0.743-1.818)	
**Clinical T stage**				
1+2	169 (84.5%)	428 (86.5%)	1.00	p=0.500
3+4	31 (15.5%)	67 (13.5%)	0.853 (0.538-1.354)	
**Pathologic T stage**				
2	113 (56.5%)	253 (51.1%)	1.00	p=0.198
3+4	87 (43.5%)	242 (48.9%)	1.242 (0.893-1.729)	
**Pathologic N stage**				
N0	188 (94.0%)	447 (90.3%)	1.00	p=0.116
N1	12 (6.0%)	48 (9.7%)	1.682 (0.874-3.239)	
**Seminal vesicle invasion**				
No	163 (81.5%)	384 (77.6%)	1.00	p=0.253
Yes	37 (18.5%)	111 (22.4%)	1.273 (0.841-1.928)	
**Perineural invasion**				
No	63 (31.5%)	121 (24.4%)	1.00	p=0.056
Yes	137 (68.5%)	374 (75.6%)	1.421 (0.990-2.041)	
**Lymphovascular invasion**				
No	173 (86.5%)	412 (83.2%)	1.00	p=0.285
Yes	27 (13.5%)	83 (16.8%)	1.291 (0.807-2.064)	
**Biochemical recurrence**				
No	148 (74.0%)	327 (66.1%)	1.00	p=0.042*
Yes	52 (26.0%)	168 (33.9%)	1.462 (1.013-2.110)	
**D''Amico classification**				
Intermediate risk	103 (51.5%)	245 (49.5%)	1.00	p=0.632
High risk	97 (48.5%)	250 (50.5%)	1.084 (0.780-1.505)	

The ORs with analyzed by their 95% CIs were estimated by logistic regression models. * *p* value < 0.05 as statistically significant.

**Table 4:** Odds ratio (OR) and 95% confidence interval (CI) of clinical status and *visfatin* rs61330082 genotypic frequencies in 364 patients with prostate cancer with PSA concentration > 10 ng/mL.

Variable	Genotypic frequencies			
rs61330082	GG (N=110)	GA+AA (N=254)	OR (95% CI)	p value
**Pathologic Gleason grade group**				
1+2+3	88 (80.0%)	188 (74.0%)	1.00	p=0.221
4+5	22 (20.0%)	66 (26.0%)	1.404 (0.814-2.422)	
**Clinical T stage**				
1+2	87 (79.1%)	199 (79.3%)	1.00	p=0.874
3+4	23 (20.9%)	55 (21.7%)	1.045 (0.604-1.808)	
**Pathologic T stage**				
2	50 (45.5%)	89 (35.0%)	1.00	p=0.060
3+4	60 (54.5%)	165 (65.0%)	1.545 (0.980-2.436)	
**Pathologic N stage**				
N0	103 (93.6%)	216 (85.0%)	**1.00**	p=0.022*
N1	7 (6.4%)	38 (15.0%)	**2.589 (1.118-5.994)**	
**Seminal vesicle invasion**				
No	81 (73.6%)	166 (65.4%)	1.00	p=0.120
Yes	29 (26.4%)	88 (34.6%)	1.481 (0.901-2.433)	
**Perineural invasion**				
No	30 (27.3%)	41 (16.1%)	**1.00**	p=0.014*
Yes	80 (72.7%)	213 (83.9%)	**1.948 (1.139-3.331)**	
**Lymphovascular invasion**				
No	93 (84.5%)	187 (73.6%)	**1.00**	p=0.023*
Yes	17 (15.5%)	67 (26.4%)	**1.960 (1.089-3.527)**	
**Biochemical recurrence**				
No	74 (67.3%)	137 (53.9%)	**1.00**	p=0.018*
Yes	36 (32.7%)	117 (46.1%)	**1.755 (1.099-2.805)**	
**D'Amico classification**				
Intermediate risk	40 (36.4%)	69 (27.2%)	1.00	p=0.078
High risk	70 (63.6%)	185 (72.8%)	1.532 (0.951-2.468)	

The ORs with analyzed by their 95% CIs were estimated by logistic regression models. * *p* value < 0.05 as statistically significant.

**Table 5:** Odds ratio (OR) and 95% confidence interval (CI) of clinical status and *visfatin* rs11977021 genotypic frequencies in 364 patients with prostate cancer with PSA concentration > 10 ng/mL.

Variable	Genotypic frequencies			
**rs11977021**	**CC (N=111)**	**CT+TT (N=253)**	**OR (95% CI)**	**p value**
**Pathologic Gleason grade group**				
1+2+3	88 (79.3%)	188 (74.3%)	1.00	p=0.308
4+5	23 (20.7%)	65 (25.7%)	1.323 (0.772-2.267)	
**Clinical T stage**				
1+2	88 (79.3%)	198 (78.3%)	1.00	p=0.827
3+4	23 (20.7%)	55 (21.7%)	1.063 (0.615-1.838)	
**Pathologic T stage**				
2	48 (43.2%)	91 (36.0%)	1.00	p=0.188
3+4	63 (56.8%)	162 (64.0%)	1.356 (0.861-2.138)	
**Pathologic N stage**				
N0	103 (92.8%)	216 (85.4%)	1.00	p=0.057
N1	8 (7.2%)	37 (14.6%)	2.205 (0.992-4.905)	
**Seminal vesicle invasion**				
No	80 (72.1%)	167 (66.0%)	1.00	p=0.254
Yes	31 (27.9%)	86 (34.0%)	1.329 (0.815-2.168)	
**Perineural invasion**				
No	29 (26.1%)	42 (16.6%)	**1.00**	p=0.035*
Yes	82 (73.9%)	211 (83.4%)	**1.777 (1.038-3.041)**	
**Lymphovascular invasion**				
No	93 (83.8%)	187 (73.9%)	**1.00**	p=0.040*
Yes	18 (16.2%)	66 (26.1%)	**1.824 (1.024-3.249)**	
**Biochemical recurrence**				
No	73 (65.8%)	138 (54.5%)	**1.00**	p=0.046*
Yes	38 (34.2%)	115 (45.5%)	**1.601 (1.007-2.545)**	
**D'Amico classification**				
Intermediate risk	39 (35.1%)	70 (27.7%)	1.00	p=0.152
High risk	72 (64.9%)	183 (72.3%)	1.416 (0.879-2.282)	

The ORs with analyzed by their 95% CIs were estimated by logistic regression models. * p value < 0.05 as statistically significant.
